# Role of Stem Cells in the Ovarian Tissue Cryopreservation and Transplantation for Fertility Preservation

**DOI:** 10.3390/ijms222212482

**Published:** 2021-11-19

**Authors:** Jeong Min Kim, Seongmin Kim, Sanghoon Lee

**Affiliations:** 1 Department of Obstetrics and Gynecology, Korea University College of Medicine, Seoul 02841, Korea; mollykim918@gmail.com; 2 Gynecologic Cancer Center, CHA Ilsan Medical Center, CHA University College of Medicine, Goyang-si 10414, Korea; naiad515@gmail.com

**Keywords:** stem cell, ovarian tissue, cryopreservation, transplantation, fertility preservation, chemotherapy

## Abstract

Although the cancer survival rate has increased, cancer treatments, including chemotherapy and radiotherapy, can cause ovarian failure and infertility in women of reproductive age. Preserving fertility throughout cancer treatment is critical for maintaining quality of life. Fertility experts should propose individualized fertility preservation methods based on the patient’s marital status, pubertal status, partner status, and the urgency of treatment. Widely practiced fertility preservation methods, including ovarian transposition and embryo and oocyte cryopreservation, are inappropriate for prepubertal girls or those needing urgent initiation of cancer treatment. Ovarian tissue cryopreservation and transplantation, an emerging new technology, may be a solution for these cancer patients. The use of stem cells in ovarian tissue cryopreservation and transplantation increases oxygenation, angiogenesis, and follicle survival rates. This review discusses the recent advances in ovarian tissue cryopreservation and transplantation with special focus on the use of stem cells to improve fertilization techniques.

## 1. Introduction

A large number of young women of reproductive age are diagnosed with cancer each year worldwide, many of whom require fertility preservation prior to cancer treatments such as chemotherapy and radiotherapy. In 2020, approximately 1,806,590 new patients were diagnosed with cancer in the United States, of whom 912,930 were women [1]. As the incidence rate of cancer is increasing in young reproductive-aged women aged from 15 to 39 years [2], the emerging issue of fertility preservation is crucial.

Cancer treatments using chemotherapy and radiation therapy increase patient survival rates; however, these treatments can cause the loss of ovarian function, eventually leading to primary ovarian insufficiency, thus precluding pregnancy. Alkylating agents such as cyclophosphamide (an anticancer drug for lymphoma, multiple myeloma, leukemia, and breast cancer) are known to be severely gonadotoxic [3,4], and their use results in follicular depletion and increases the risk of iatrogenic primary ovarian insufficiency [5,6]. Not only cancer patients but also patients undergoing surgery for benign conditions, such as endometriosis, are known to experience fertility loss due to treatment [7,8]. Therefore, the need for fertility preservation for these reproductive-aged patients is emerging, and early referrals to fertility experts for fertility preservation is essential [9,10,11].

Ovarian transposition and embryo and oocyte cryopreservation are widely adopted techniques for preserving the fertility of cancer patients. However, embryo and oocyte cryopreservation are not suitable for prepubertal cancer patients who need urgent chemo-therapy and do not have enough time for ovarian stimulation [12]. Therefore, ovarian tissue cryopreservation and transplantation is an option for prepubertal cancer patients who need to preserve fertility prior to chemotherapy or radiation therapy in circumstances that are not suitable for ovarian stimulation. More than 130 live births through orthotopic transplantation of ovarian tissue have been reported [13,14,15,16], and the cumulative success rate of ovarian tissue is approximately 20–40% [6,17]. Ischemic follicle loss occurs after ovarian tissue transplantation.

To improve the success rate, the use of stem cells to prevent follicle loss has been investigated, and its efficacy is promising. This review discusses the recent advances in ovarian tissue cryopreservation and transplantation with special focus on the use of stem cells to improve fertilization techniques.

## 2. Identification and Isolation of Ovarian Stem Cell

In human reproductive biology, it has been considered a doctrine that the reserve of oocytes is fixed at birth and cannot be renewed, as established by Green and Zuckerman in 1951 [18]. Although males retain the ability to produce germline stem cells (GSCs), females were thought not to produce oocytes after birth [19]. The number of oocytes de-creases throughout life, and follicle exhaustion eventually leads to menopause [20].

**Figure 1:**
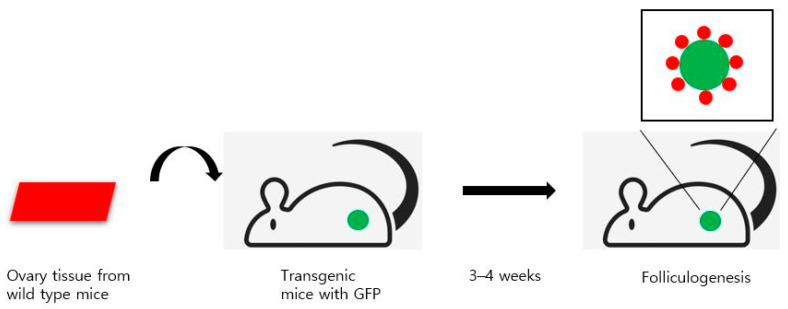
The experiment using transgenic mice with GFP by Tilly et al.: folliculogenesis in postnatal life. Wild type ovaries grafted into transgenic female mice with green fluorescent protein (GFP) were infiltrated with GFP-positive germ cells that formed follicles. After 3 to 4 weeks, GFP-positive oocytes were surrounded by wild-type granulosa cells, indicating that ovarian stem cells from the GFP mice had initiated folliculogenesis.

However, after 50 years, Tilly et al. challenged this theory in 2004 by reporting the existence of mitotically active GSCs in postnatal ovaries in mice [21]. The experiment showed that adult mouse ovaries possess mitotically active germ cells that regenerate the follicle reserve in adulthood. To understand germ cell dynamics in female mammals, the number of follicles was counted in mouse ovaries. They found that the degeneration of atretic follicles results in the depletion of primordial follicle reserve by young adulthood, and the GSCs represent the source of the new oocytes. Depending on the rate of oocyte degeneration, mitotically active germ cells continuously replenish the pool of follicles. When prepubertal female mice were treated with busulfan, a gonadotoxic alkylating chemo-therapeutic drug, the primordial follicle reserve was eradicated. After elimination, the meiotic marker synaptonemal complex protein 3 was observed. In another set of experiments, wild-type ovaries grafted into transgenic female mice with green fluorescent protein (GFP) were infiltrated with GFP-positive germ cells that formed follicles. GFP-positive oocytes were surrounded by wild-type granulosa cells, indicating that ovarian stem cells from the GFP mice had initiated folliculogenesis. Thus, the authors demonstrated that mammalian female GSCs regenerate the follicle reserve in adult life (Figure 1).

In 2005, Tilly et al. proposed that bone marrow might be a source of germ cells in postnatal mouse ovaries. They hypothesized that bone marrow-derived germ cells could be transported by peripheral blood circulation to generate oogenesis. However, subsequent studies showed that transplantation of bone marrow cells did not directly contribute to oocyte formation, as they belong to the hematopoietic lineage [22,23,24]. Ovarian GSCs have been identified in prepubertal rats and pigs, and they were also isolated from reproductive-aged women in 2012 [25,26,27]. Female germline cells were injected into human ovarian cortical tissues, and after xenotransplantation into immunodeficient female mice, follicles containing oocytes were identified [28]. This study investigated whether stem cells isolated from human ovaries can differentiate into oocytes in vitro and in vivo and determined that proliferative GSCs exist in postnatal mammalian ovaries.

Postnatal oogenesis in ovaries of reproductive age has been confirmed, and postnatal oogenesis has been used to produce offspring [29,30,31]. In many laboratories around the world, the existence of ovarian GSCs have been proved, and now there is no doubt that the old doctrine of ovarian reserve endowed at birth should be replaced with a new doctrine of ovarian GSCs [31].

The production of stem cells by oocytes could be the key to solving infertility [32]. Stem cells purified from ovarian GSCs may be an option for reproductive-aged cancer patients who are subjected to iatrogenic primary ovarian insufficiency, particularly prepubertal girls. The fertilization of stem cell-derived oocytes provides a new direction in the field of artificial reproductive techniques, which may be applied in the near future. Stem cells may be implanted in the patient’s ovary or cultured into oocytes for in vitro fertilization [33,34,35].

However, due to several restrictions and little clinical research conducted on purifying ovarian stem cells for reproduction, it has not yet been clinically applied [22,36]. Moreover, their scarcity is another problematic issue. White et al. reported that ovarian stem cells constitute 0.014% of all cells in mouse ovaries [28]. As age increases, the number of ovarian stem cells decreases from 2% to 1% in neonatal mouse ovaries and to 0.05% in adults [37]. In addition, the field is challenged by ethical issues, and inadequate isolation and differentiation protocols make it difficult to generate oocytes directly from GSCs [31]. Furthermore, the epigenetic status of embryogenesis in postnatal mammalian gonads needs to be investigated [38]. With more research, the isolation of ovarian stem cells might become useful to increase the outcomes of ovarian tissue cryopreservation and transplantation [35,39,40].

## 3. Ovarian Tissue Cryopreservation and Transplantation

Ovarian tissue cryopreservation and transplantation are promising alternative options for prepubertal girls or young women who need urgent chemotherapy and do not have enough time for ovarian stimulation. In 2004, Donnez et al. successfully reported a live birth after orthotopic transplantation of cryopreserved ovarian tissue. The ovarian cortex tissue of a woman with Hodgkin lymphoma stage IV was preserved prior to chemotherapy. In 2003, after chemotherapy treatment, the thawed ovarian tissue was transplanted laparoscopically, and the follicle was observed five months after reimplantation. Other parameters, including basal body temperature, menstrual cycles, and hormone concentrations, showed that she was experiencing regular menstrual cycles. After 11 months, intrauterine pregnancy was confirmed, and she gave birth to her child [41]. The advantages of ovarian tissue transplantation include not only preservation of fertility, but also restoration of endocrine function in young women after cancer treatment [42,43]. However, the success rate is decreased by the lack of oxygenation and angiogenesis.

The duration of graft function depends on several factors, including the age at the time of cryopreservation, baseline ovarian reserve, history of cancer treatment, ovarian tissue preparation techniques, freeze-thawing protocols, amount of grafted cortical tissue, transplantation sites and techniques, degree of ischemia after transplantation, and the number of follicles in the ovarian grafts. As such, the use of growth factors or stem cells is considered to increase efficiency [44,45]. As a result of ischemia, the graft loses more than two-thirds of the primordial follicles during revascularization [46]. Various methods have been devised to improve ovarian tissue cryopreservation and transplantation outcomes. The use of anti-apoptotic agents, recombinant AMH, and growth factors has been shown to increase success rates. Experiments have been conducted to determine whether slow freezing or vitrification is superior for cryopreservation [32].

The use of anti-apoptotic agents, such as sphingosine-1-phosphate (S1P) and Z-VAD-FMK, has been shown to be effective in preserving primordial follicles and preventing tissue damage during ovarian tissue cryopreservation and xenotransplantation [47]. S1P is a natural inhibitor of ceramide, and it has been shown to prevent oocyte loss and infertility [48,49]. According to Morita et al., S1P in vivo treatment completely prevented radiation-induced oocyte loss in adult wild-type female mice. The report showed that sphingomyeline pathways regulate the death of oocytes [48]. Other articles showed that S1P reduces primordial follicle loss in human ovarian tissue xenografted in mice [50,51], and it is also reported to prevent loss of primordial follicles during the vitrification and slow freezing of mouse ovarian tissue grafts [52,53]. The ovaries were pretreated with S1P before vitrification of cryopreservation, and AMH levels were compared. The number of primordial follicles were shown to be much higher in S1P-treated groups compared to the control group. The use of S1P in the graft enhances fertility preservation by protecting the vitrified ovarian graft from ischemic reperfusion injury. The use of anti-apoptotic molecule exposure of oocytes to chemotherapeutic agents results in apoptosis leading to cell death [51]. S1P seemed to prevent pathways involving the apoptosis of oocytes.

Other molecules to minimize ovarian damage caused by anticancer treatment include circumin and capsaicin to account for cyclophosphamide-induced ovarian premature ovarian failure [54]. The use of imatinib (Gleevec) has been reported to minimize follicle depletion in mice caused by cisplatin chemotherapy. However, the use of imatinib, a targeting receptor associated tyrosine kinase is still debated for the application of fertility preservation in patients undergoing cytotoxic treatment.

Along with an effort to minimize follicle loss during ovarian tissue cryopreservation and transplantation, methods for preserving fertility during cytotoxic chemotherapy have been applied to protect existing oocytes. The use of gonadotropin releasing hormone (GnRH) agonists reduce the loss of ovarian follicles caused by cyclophosphamide exposure of cancer treatment [55]. This is related to the protective effects of GnRH agonists by downregulating the secretion of follicular stimulating hormone and pituitary luteinizing hormone. The use of GnRH agonist minimized ovarian damage, and the resumption of menstruation cycles has been observed. Previous studies have shown that ovarian suppression with GnRH agonists during chemotherapy protects ovarian function in young cancer patients treated for lymphoma, breast cancer, and other diseases [56,57,58]. However, whether GnRH agonists actually improved pregnancy outcomes for cancer patients after cytotoxic treatment is questionable. The role of GnRH analogs could result from two possible mechanisms [57,59,60]. First, the sensitivity of the primordial follicles entering the growing pool to gonad toxicity could be decreased by the administration of GnRH agonist. The second mechanism constitutes the direct anti-apoptotic effect of GnRH agonists on ovarian germline stem cells. The cotreatment using GnRH agonists, in combination with oocyte or embryo cryopreservation, may be a good option [61].

These currently investigated agents, as well as stem cells used to aid ovarian tissue cryopreservation and transplantation, have been researched to minimize follicle loss and aid follicle maturation (Table 1). Among the cryoprotectants used for slow freezing, ethylene glycol showed the highest follicle survival rate compared to dimethyl sulfoxide, propylene glycol, and glycerol [32]. The use of recombinant AMH, VEGF, and FGF2 has been shown to reduce initial follicle loss and improve revascularization [62,63,64]. Some studies reported that the slow-freezing method yielded better results than vitrification, but it remains controversial [65]. Despite these improved methods, post-graft ischemia remains an issue. Innovative new technology is needed for ovarian cryopreservation and transplantation, and stem cells may fulfill this role.

**Figure 2:**
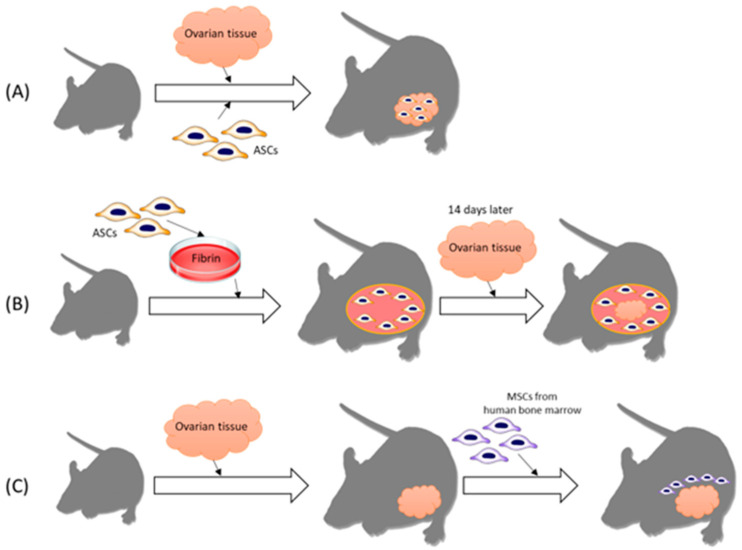
Several methods for utilizing human stem cells for ovarian tissue transplantation. (**A**) Ovarian tissue was implanted on the retroperitoneum of the abdomen. Then ASCs were injected directly into the center of the transplanted ovarian tissue. (**B**) Fibrin scaffold was prepared with ASCs and implanted on the inner peritoneal surface of the mouse. Fourteen days later, ovarian tissue was placed between the fibrin and peritoneum. Increased oxygenation around implanted ovarian tissue was seen. (**C**) Ovarian tissue was implanted into the subcutaneous area of the abdomen. MSCs obtained from human bone marrow were placed under the graft with growth factor-reduced Matrigel. MSCs may help neovascularization and blood perfusion of transplanted grafts. ASC, adipose tissue-derived stem cell; MSC, mesenchymal stem cell.

There are several methods to utilize human stem cells for ovarian tissue transplantation (Figure 2). Recent studies have investigated the effects of adipose tissue-derived stem cells (ASCs) in a mouse model. These experiments show the effect of stem cells in ovary tissue transplantation by means of angiogenesis, follicle maturation, oxygenation, and anti-apoptosis. Manavella et al. reported that ASCs boost vascularization in grafted ovarian tissue by secreting growth factors and differentiating into endothelial cell lineages. Compared to the control group, the use of stem cells resulted in increased levels of VEGF and FGF2, which are proangiogenic factors [66]. In another experiment conducted by Manavella et al., ASCs at high concentrations in a fibrin implant showed enhanced neovascularization [51]. In a mouse model of peritoneal grafts using low and high doses of ASCs, oxygenation levels were checked in vivo using electron paramagnetic resonance (EPR) oximetry. Regardless of the dosage of ASCs, the use of ASCs led to an increase in oxygenation, which correlated with implant vascularization. The experiment implicated the use of EPR oximetry as a way to evaluate angiogenesis in ovarian transplantation, and the researchers concluded that preparing a grafting site with a high concentration of ASCs in the fibrin scaffold enhanced vascularization [67].

ASCs, autologous mesenchymal stem cells (MSCs), are known to differentiate into multiple lineages. ASCs are easily obtainable and are used in the graft site of ovary transplantation [68]. Many studies have demonstrated the capability of ASCs to enhance angiogenesis by secreting growth factors [69,70,71]. It is known that hypoxia persists for the first five days post-transplantation and is the main cause of follicle survival [72]. ASCs have been shown to decrease post-transplantation ischemic injury.

However, direct injection of stem cells into the ovarian tissue might not be appropriate for follicle proliferation according to Damous et al. A single dose of rat ASCs was injected into the bilateral cryopreserved ovaries of adult female rats immediately after an autologous transplant. Although the long-term outcome has not been investigated, ASC-treated grafts were impaired, with diffuse atrophy and increased apoptosis. The authors explained that the direct injection of ASCs may have overstimulated the intrinsic inflammatory response in an environment already under adverse hypoxic conditions [73]. This study determined that the direct injection of ASCs did not improve follicular survival, and instead increased apoptosis level.

The effect of injecting bone marrow-derived MSCs was investigated by Xia et al. [74]. Human ovarian tissues were transplanted with human MSCs into the heterotopic site at the subcutaneous area of the abdominal wall of the mice. MSCs transplanted with ovarian tissues showed stimulated neovascularization, enhanced expression of VEGF, FGF2, and angiogenin, and increased functional blood perfusion. Decreased apoptotic rates of primordial follicles and improved follicle survival were observed. Compared to the use of FGF2, which also showed an improvement in angiogenesis, MSCs showed long-lasting angiogenesis in ovarian cortex transplantation as they established an angiogenic microenvironment by persistently secreting biomolecules in the graft [74,75]. Furthermore, angiogenin has been identified as a key MSC-secreted factor involved in follicle survival and revascularization of xenografted human ovarian tissue by enhancing angiogenesis and endothelial cell proliferation [76].

The use of ASCs in graft sites has been shown to be efficient according to Manavella et al. Frozen-thawed human ovarian tissues were transplanted in severe combined immunodeficient (SCID) mice, and the peritoneal transplantation site was loaded with ASCs two weeks prior to transplantation. Compared to the control group, the use of ASCs in the grafts resulted in higher oxygen levels on day 7, as well as a higher primordial follicle survival rate. The study showed that ASCs enhanced vascularization in the early post-grafting period, leading to increased follicle survival rates and decreased apoptosis rates [77].

**Table 1:** Articles of stem cells in ovarian tissue cryopreservation and transplantation.

Reference	Animal Model	Type of Stem Cells	Main Findings
[63]	SCID mice	Human ASCs	ASCs boosted vascularization in grafted ovarian tissue by secreting growth factor.
[64]	SCID mice	Human ASCs	The use of ASC in fibrin implant showed an increase in oxygenation.
[70]	Rat	Rat ASCs	Direct injection of ASCs into cryopreserved ovaries did not improve follicular survival.
[71]	Mice	Human MSCs from bone marrow	Human MSCs isolated from bone marrow increased the levels of VEGF, FGF2, and angiogenin, stimulated neovascularization, and increased blood perfusion of transplanted grafts.
[74]	SCID mice	Human ASCs	Prior to human ovary tissue transplantation in SCID mice, peritoneal transplantation was loaded with ASCs. Increased oxygenation, enhanced vascularization, increased primordial follicle survival rate, and decreased apoptosis rate were shown.

SCID, severe combined immunodeficient; ASCs, adipose tissue-derived stem cells; MSCs, mesenchymal stem cells.

## 4. Other Fertility Preservation Methods Using Stem Cells

### 4.1. Direct Injection of Stem Cells

Ovarian Germline Stem Cells (GSC) transferred into cancer patients after chemotherapy might preserve fertility and recover normal menstrual cycles, according to recent research. GFP-expressing ovarian GSCs were transplanted into the ovaries of adult wild-type female chemotherapy-treated mice and then differentiated into GFP-positive oocytes producing offspring with the GFP transgene [78]. This experiment, performed about a decade ago, shows how ovarian GSC transplantation can undergo oogenesis. Another research team again confirmed the outcome of GSC transplantation into adult female mice treated with chemotherapy: ovarian function was restored, and the recipients produced offspring [79]. In addition, spermatogonial stem cell transplantation into busulfan-treated mice has enabled them to produce offspring, which is equivalent to oogenesis in vivo in mice [80]. However, regenerating oogenesis in vivo in humans is still a challenging issue for preserving fertility.

### 4.2. Bone Marrow Transplantation

Bone marrow transplantation (BMT) is another usage of stem cells for fertility preservation. Bone marrow seems to be an extragonadal source of germ cells. BMT into female mice treated with busulfan partially restored oogenesis [23]. The team also showed oocyte regeneration in ataxia telangiectasia mutated gene deficient mice which are genetically incapable of oogenesis. Producing offspring through BMT in humans as peripheral blood derived oocyte is still questionable. However, recent studies show human bone marrow-derived stem cells promoting follicle development in patients with impaired ovarian functions [81]. When human bone marrow-derived stem cells were injected into mice with chemotherapy-induced ovarian damage, promotion of angiogenesis and increased production of preovulatory follicles were seen. This research presents the possibility of using autologous bone marrow-derived stem cells for preserving fertility in patients undergoing chemotherapy.

### 4.3. IVM

In vitro maturation (IVM) of primordial follicles from cryopreserved ovarian tissue is another fertility preservation method. Antral follicles are isolated from cryopreserved ovarian tissue until complete oogenesis has been achieved [82]. IVM is a promising fertility preservation method as it prevents the risk of reintroducing cancer cells back into the patient [83]. IVM of oocytes has shown lower maturation rates, especially in pre-menarche patients [84]. Stem cells could be applied to improve the outcome of IVM.

MSCs were used to increase survival and growth rate of cultured follicles. MSCs from human umbilical cord were conditioned in a medium and resulted in an increase in vessel density and a decrease in apoptosis of cultured cortical tissue [13,85]. Another experiment with MSCs derived from human endometrium showed better outcomes in the IVM of mouse preantral follicles. With the use of human menstrual blood mesenchymal stem cells, follicular growth ensued, including survival rate, diameter, antrum formation and the rate of in vitro maturation (IVM) [86]. These experiments imply that the application of stem cells might also allow improvement in follicle growth in human IVM.

## 5. Conclusions

Due to advances in cancer treatment, cancer survival rates have increased, and early referral to fertility experts for fertility preservation is critical [9]. Although embryo or oocyte cryopreservation have been considered the standard methods for fertility preservation, ovary tissue cryopreservation has been accepted as an alternative method of fertility preservation for prepubertal girls and patients who need immediate treatment [87]. The recovery of endocrine function after reimplantation is well established, and the live birth rate has been substantially increasing [88]. Along with the use of stem cells in ovarian tissue cryopreservation and transplantation, direct injection of stem cells, bone marrow transplantation, and IVM are other recent promising fertility preservation methods for young female cancer patients undergoing chemotherapy. Although further research is needed with human clinical trials prior to implementation, stem cell biology techniques combined with existing fertility preservation and restoration methods seem to be a prospective field, especially for prepubertal girls or for women who have no time for ovary stimulation.

The use of stem cells for ovarian tissue cryopreservation and transplantation has not yet been clinically implemented in humans. Further studies are required to implement these approaches for human treatment. We believe that the use of stem cells, which is still under investigation, can aid in oxygenation, follicle maturation, anti-apoptosis, and angiogenesis in ovarian tissue cryopreservation and transplantation. To prevent fertility loss in women with cancer, individualized fertility preservation strategies must consider the patient’s marital status and ovarian reserve [32,88], and the use of stem cells seems to be a promising option for improving the success rate of ovarian tissue transplantation. With the ability to isolate human ovarian stem cells, the use of ovarian stem cells for fertility preservation provides new prospects in this era of human reproduction for women with cancer.
